# High power diode laser beam welding of AA8011 aluminum alloy for enhanced mechanical performance in lightweight structures

**DOI:** 10.1038/s41598-026-41272-1

**Published:** 2026-02-26

**Authors:** Rajesh Patil, Magnus Löfstrand

**Affiliations:** School of Science and Technology, (Mechanical Engineering, Digitalized Product and Production Development), Örebro University, 701 82 Örebro, Sweden

**Keywords:** High power diode laser welding, Aluminum AA8011 alloy, Parametric optimization, Mechanical properties, Microstructure characterization, Sustainable manufacturing, Engineering, Materials science

## Abstract

Aluminum alloy AA8011 is widely utilized in automotive and lightweight engineering applications, although traditional fusion welding frequently produces faults due to its high thermal conductivity and restricted weldability. This work investigates High Power Diode Laser Beam Welding (HPDLBW) of 2 mm AA8011 sheets employing laser power (3.2–3.4 kW), welding speed (17–23 mm/s), shielding gas (20 L/min) and beam diameter (3–4 mm) built with a Taguchi L9 orthogonal array. A response surface model linked process factors to mechanical responses, including impact energy, hardness, and tensile strength. Optimal settings for multi-objective optimization (P = 3.3 kW, v = 17 mm/s, d = 3.5 mm) resulted in 110 J of impact energy, 33 HV0.5 of hardness, and 69 N/mm^2^ of tensile strength. Analysis of variance revealed that laser power accounted for up to 38.6% of the variation in weld penetration, confirms its prominent role in melt pool development, while tensile strength improved by a maximum of 14.2% under optimal conditions. Under optimum conditions, microstructural research revealed refined fusion zones, minimized intermetallic formation, and little porosity. The work shows that Taguchi-based modeling and optimization give a predictive framework for enhancing AA8011 weld performance, and it establishes HPDLBW as a dependable method for lightweight alloy joining. The study demonstrates HPDLBW as a viable and scalable joining technology for lightweight aluminum structures, with lower heat input, improved surface polish, and practical significance for the automotive and packing industries.

## Introduction

**Fig. 1:**
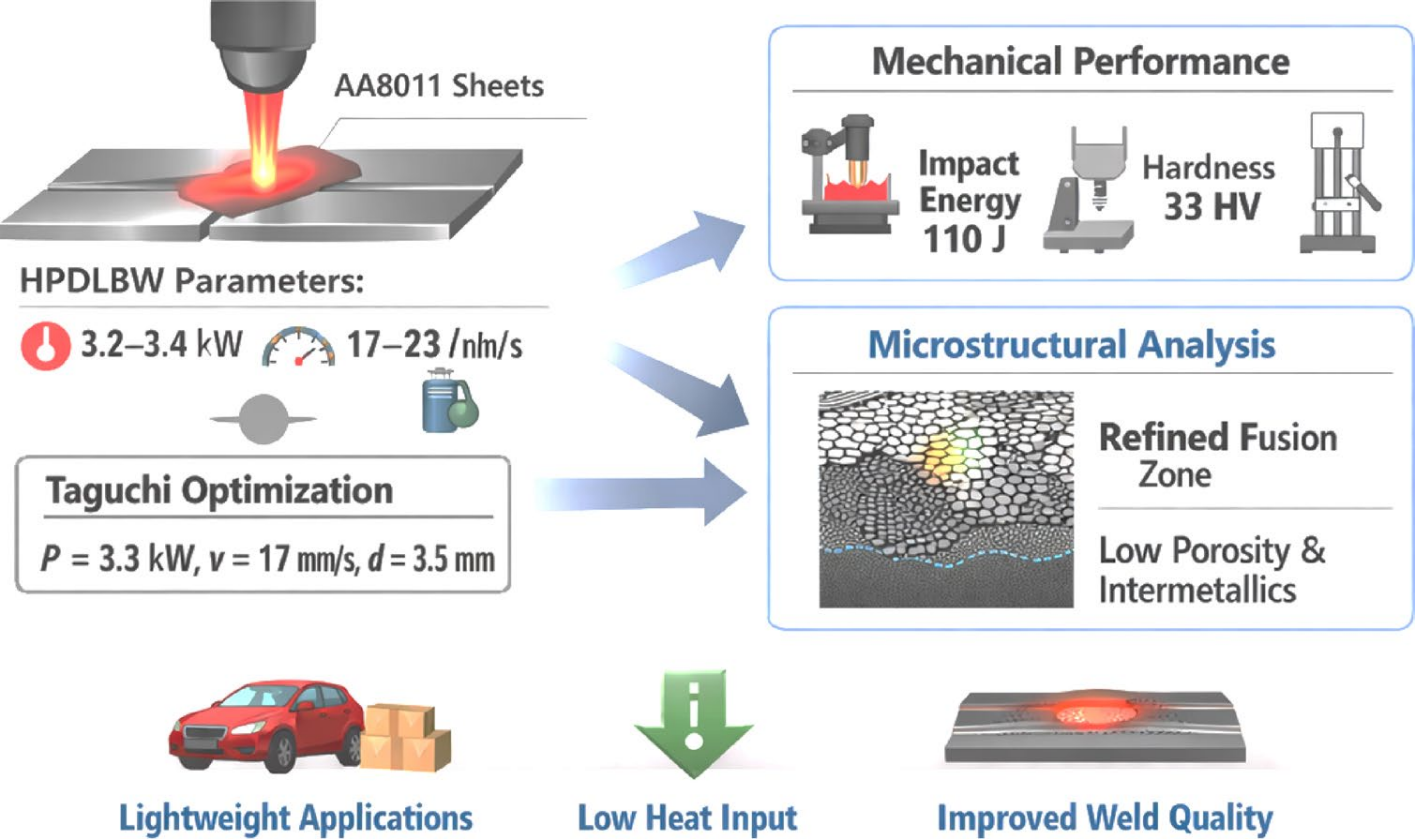
Overview of HPDLBW welding optimization for AA8011 aluminum alloy sheets.

Aluminum alloys are commonly utilized in the automotive, aerospace, HVAC, packaging, and energy industries because of their excellent strength-to-weight ratio, corrosion resistance, formability, and recyclability^1,2^. AA8011 is widely used in thin foils and sheets, where weight reduction immediately leads to improved fuel efficiency, lower emissions, and enhanced payload capacity. However, welding AA8011 remains difficult due to its high thermal conductivity, unstable oxide layer, and susceptibility to deformation, porosity, and other problems during fusion welding^3,4^. Conventional methods, such as TIG and MIG welding, frequently result in excessive heat input, high distortion, and increased defect formation, whereas solid-state techniques, particularly friction stir welding, can improve joint integrity, though their performance is heavily dependent on tool design, rotational speed, and traverse speed^5,6^. The optimization of tool geometry and process parameters has been proven to reduce grain coarsening and improve weld stability, particularly in dissimilar material combinations such as AA8011-AA6062^7–11^. Fusion-based alternatives, such as hybrid laser-arc welding and cold metal transfer, have also been investigated, but they typically involve trade-offs between heat-input control, defect formation, and industrial feasibility^12–16^, and low-distortion, automation-ready solutions for thin AA8011 sheets remain scarce^17–20^. High-Power Diode Laser Beam Welding (HPDLBW) is a promising solution with localized heat input, narrow heat-affected zones, high electrical-to-optical efficiency (~ 40%), fiber-coupled and micro-channel-cooled modules, and strong absorption in reflective materials like aluminum and stainless steel^21–24^. These capabilities allow for conduction-mode welding with minimal filler material, reduced porosity, and precise dimensional control, making HPDLBW ideal for automated manufacturing environments, robotic production cells, EV battery modules, automotive thin-sheet assemblies, HVAC systems, and precision packaging applications^25–31^. Advanced approaches, such as RNN, ANN, and SVM algorithms, have been effectively used for weld fault identification and classification, highlighting the potential for process monitoring and quality assurance in high-power laser welding^32–40,41^. Despite advances, systematic research on HPDLBW of thin AA8011 sheets (≤ 2 mm) is scarce, particularly regarding process window mapping of laser power, welding speed, and beam diameter; conduction-mode fusion behavior; microstructural evolution in the parent metal, heat-affected zone, and weld metal; defect formation mechanisms; and integrated mechanical responses including tensile strength, impact toughness, and microhardness. To fill these gaps, the current work looks into the weldability of 2 mm AA8011 sheets using HPDLBW and compares the effects of laser power, welding speed, beam diameter, and shielding parameters on weld quality. A Taguchi L9 experimental design, together with response surface regression and multi-objective optimization, is used to establish the optimal process parameters and predictive models for weld performance, as shown in Fig. 1.

## Experimental investigation of AA8011 weld joints

The welding trials were performed utilizing a High-Power Diode Laser Beam Welding (HPDLBW) system with a maximum output power of 3.4 kW. The system operates in the wavelength range of 808–980 nm and employs fiber-coupled beam delivery with a 600 µm core diameter. The laser beam was concentrated to a 3–4 mm spot diameter, and argon shielding gas was delivered at a constant flow rate of 20 L/min to reduce oxidation during welding. AA8011 aluminum alloy sheets with a thickness of 2 mm and dimensions of 100 × 50 mm were butt-welded lengthwise. The tests were planned using a Taguchi L9 orthogonal array, with laser power (3.2–3.4 kW), welding speed (17–23 mm/s), and beam diameter (3–4 mm) as the principal control parameters, with the shielding gas flow rate held constant at 20 L/min. Mechanical characterization of the welded joints was performed using a Vickers microhardness tester with a load of 500 g and a dwell time of 15 s, a universal tensile testing machine in accordance with ASTM E8/E8M at a crosshead speed of 0.033 mm/s, and an impact testing machine in accordance with ASTM E23 with a 10 J pendulum. SEM (JEOL JSM-6510, working at 15 kV) was used to investigate dendritic morphology and intermetallic phase development in the weld and heat-affected zones. This experimental setup allowed for a systematic assessment of the impacts of HPDLBW process parameters on weld shape, microstructural evolution, and mechanical performance of 2 mm AA8011 aluminum alloy sheets under conduction-mode welding circumstances.

### Design of experiments and process parameters

The design of experiments (DoE) is used to investigate the relationship between many input factors known as process parameters and key outcome variables such as depth of penetration and bead width. DoE is a comprehensive data gathering and analysis tool that may be utilized in a wide range of experimental settings. The major influencing parameters on the depth of penetration are welding power, speed, beam diameter and shielding gas are shown in Table 1. The experiments were designed using Taguchi L9 (4 parameters × 3 levels) orthogonal arrays. A 2 mm sheet thickness is used. The butt joints are employed, and the edges are formed with square butts spaced 1.5 mm apart to maintain the center-to-center distance between adjacent weld seams was kept constant at 1.5 mm, guaranteeing proper overlap and equal heat distribution over the weld zone. Fixtures are crucial for HPDLBW welding thin aluminum sheets, which warp rapidly because to their high thermal conductivity and low rigidity. The copper base plate fixture material is preferred for heat dissipation and mechanical screw clamp hold-downs along both sheet edges. The HPDLBW used in this work operated in continuous-wave mode with a top-hat beam profile, resulting in homogeneous energy distribution across a 3–4 mm beam diameter. This profile provided stable melting, reduced localized overheating, and generated smooth, consistent weld beads on the 2 mm AA8011 sheets. The beam was focused at or slightly below the surface to maximize energy coupling and penetration stability. Table 2 illustrate the matrix of process variables for aluminum materials selected based on DoE. The design levels are − 1, 0, and + 1.The nine expt. specimen were chosen for weld inspection. Welding power, speed, beam diameter, and shielding gas are the most critical process parameters for depth of penetration and bead width in expt. specimen.

**Table 1:** Process variables and levels.

Parameters	Set value		
	− 1	0	+ 1
Sheet thickness (mm)		2	
Welding power (kW)	3.2	3.3	3.4
Welding speed (mm/ sec)	17	20	23
Beam diameter (mm)	3	3.5	4

**Table 2:** Design matrix with process variables.

Expt. No	Welding power	Welding speed	Beam diameter	Sheet thickness
1	3.2	17	3	2
2	3.3	20	3.5	2
3	3.4	23	4	2
4	3.2	20	4	2
5	3.3	23	3	2
6	3.4	17	3.5	2
7	3.2	23	3.5	2
8	3.3	17	4	2
9	3.4	20	3	2

### Chemical and mechanical properties

The composition of the alloy under research is in line with common compositions reported for aluminum alloys used in automotive and aerospace applications and includes 0.6–0.65% Si, 0.8% Fe, 0.2% Cu, 0.05% Mn, 0.05% Mg, 0.001% Cr, 0.03% Zn, and 0.03% Ti. Its density of 2689 kg/m^3^ and tensile strength of about 110 MPa are in line with figures previously reported for lightweight structural alloys. Additionally, the alloy has a melting point of 660 °C and a thermal conductivity of 237 W/m K, which are like values reported in the literature for aluminum alloys that conduct heat. Its hardness of roughly 60 HRB makes it suitable for welding and shaping in aerospace and automotive applications.

### Depth of penetration

Laser welding experiments on 2 mm AA8011 aluminium sheets were carried out utilizing nine distinct combinations of laser power, welding speed, and beam diameter based on the Taguchi L9 design to completely assess weld quality. Welded specimens were first inspected on the top and bottom surfaces for apparent flaws such as porosity, fractures, and partial fusion. A macroscopic evaluation of the weld bead geometry gave an initial assessment of the influence of laser parameters on weld quality, which was then utilized to guide the selection of ideal welding settings.

To evaluate weld penetration in greater detail, specimens were transversely sectioned across the weld bead and prepared for metallographic investigation. The cross-sectional surfaces were polished successively with 150, 200, 400, 600, 800, and 1000 grit polishing sheets, then electrolytically etched in accordance with ASTM G48 to disclose the fusion zone, heat-affected zone (HAZ), and weld boundaries. Cross-sectional images were captured using a stereo metallurgical microscope at 10× magnification. Image analysis software (e.g., ImageJ) was used to quantify weld bead geometry, such as depth of penetration, bead width, and HAZ dimensions. At least three independent measurements were taken for each specimen, ensuring repeatability and reproducibility. Results are provided as mean ± standard deviation. High-magnification pictures (31.1×) were used to evaluate microstructural characteristics and validate fusion depth.

This combination approach, which incorporates macroscopic top/bottom views, cross-sectional metallography, and quantitative image analysis, enables the accurate, reproducible, and systematic evaluation of weld quality. It emphasizes the importance of laser power, welding speed, and beam diameter on depth of penetration, bead shape, and microstructural properties, giving a solid foundation for optimizing HPDLBW parameters for thin AA8011 sheets.

### Hardness test

The hardness of the welded junction was assessed in line with IS 1608:2005 standards, which establish the Vickers hardness test method for metallic materials. The test was carried out on three different parts of the specimen: the Parent Metal (PM), Heat-Affected Zone (HAZ), and Weld Metal (WM), each representing a different metallurgical condition resulting from the heat cycle of welding. Multiple indentations were performed on a 500 g specimen to acquire statistically meaningful results. Vickers microhardness measurements were taken on the welded AA8011 aluminum sheets using a microhardness tester. Specimens were sectioned perpendicular to the weld seam, mounted in epoxy, and polished using normal metallographic processes. This included grinding with 400–2000 grit papers and final polishing with 1 µm diamond paste to provide a smooth, scratch-free surface. Measurements were taken with a steady load of 500 g (HV0.5)and a dwell time of 15 s. Indentations were produced over three areas of the weld: the base metal (BM), the heat-affected zone (HAZ), and the weld zone (WZ), with a minimum distance of 2.5 times the indentation diagonal to prevent interference from neighboring locations. Three to five indentations were obtained per region, and the Vickers hardness number (HV0.5) was derived automatically using the measured diagonal lengths. Each region’s average hardness and standard deviation were recorded. The same operator performed all tests, and the device was calibrated using a standard reference block to assure accuracy and consistency. This methodology enabled a quantitative assessment of the effect of welding factors such as laser power, welding speed, and beam diameter on the mechanical properties of the welded joint.

### Impact test

The impact toughness of the laser-welded aluminium sheets was measured using a Charpy impact tester in accordance with ASTM E23. Standard full-size Charpy specimens (10 mm × 10 mm × 55 mm) couldn’t be made due to the sheet thickness constraint of 2 mm. Sub-size Charpy V-notch specimens were machined from welded sheets with dimensions of 55 mm × 10 mm × 2 mm. A 2 mm-deep V-notch was created in the center of the specimen, perpendicular to the welding direction, allowing crack propagation through the weld bead. All experiments were carried out at ambient temperature (23 °C). The absorbed impact energy was measured in joules. Because sub-size specimens were utilized, the recorded impact energy values were normalized with respect to the specimen’s cross-sectional area at the notch to facilitate comparison with standard results, as per ASTM E23 standards for reduced-thickness specimens. The impact toughness of samples generated under various welding settings was examined, including laser power of 3.2–3.4 kW, welding speed of 17–23 mm/s, and laser beam width of 3–4 mm. Each condition was tested three times, and the average absorbed energy (J) was recorded. The notch arrangement facilitated fracture propagation through the weld metal and heat-affected zone, allowing for an accurate evaluation of the weld region’s toughness.

### Tensile test

Tensile testing was carried out on sub-sized flat specimens machined from the welded joints and parent material. All tests were conducted on a universal tensile testing machine with a load capacity significantly higher than the recorded ultimate loads (≥ 10 kN). For quasi-static testing, a constant crosshead speed of 2 mm·min^−1^ was used. Nine expt. specimen of material were evaluated for tensile strength in accordance with IS 1608:2005, with specimen dimensions and results recorded. The specimens varied in thickness from 1.90 to 2 mm and breadth from 19.80 to 20.10 mm, with a cross-sectional area of 38.00–40.20 mm^2^. The gauge length for all specimens remained the same at 60.00 mm. Each specimen had a gauge length of 60.00 mm. Where applicable, the testing followed conventional tensile test practice (ASTM E8/E8M for the main dataset and IS 1608:2005 for expt. specimen 6–9 as specified). The ultimate tensile strength (UTS), yield strength, and elongation of the specimens were measured in the appropriate units. Stress levels are provided in N/mm^2^, whereas applied force during testing is indicated in kilonewtons (kN). The specimens’ elongation or strain is measured in percentage (%) of their initial gauge length.

*Variability and error analysis* were conducted in triplicate for essential data such as grain size, microhardness, tensile strength, and impact energy to assure reproducibility. The presented results are expressed as mean ± standard deviation, which provides an approximation of the experimental variability. The weld metal had an average grain size of 5.8 ± 0.7 μm, as per ASTM E112-13 standards. Where relevant, the measurement resolution or detection limits of the devices were taken into account to guarantee accurate and reliable data reporting. This approach gives a systematic depiction of variability, increasing confidence in the experimental results.

## Mathematical modeling and optimization

A mathematical response surface model (RSM) was created to establish a quantifiable relationship between the HPDLBW parameters and the mechanical performance of the AA8011 after welding. The independent variables used were laser power (P), welding speed (v), and laser spot diameter (d), while the essential responses were impact energy (E), hardness (H), and ultimate tensile strength (S).

### Model of governing heat input

The net heat input per unit length of laser beam welding determines the shape of the melt pool and the ensuing mechanical performance. The amount of heat absorbed per unit length for conduction-mode HPDLBW is:

1 $$Q = \frac{\eta P}{{v}}$$

where Q net heat input per unit length (J/mm), η absorption efficiency (≈ 0.85 for Al at 940 nm), $P$ laser power (W), $v$ welding speed (mm/s).

In Eq. (1), the absorption efficiency (η) denotes the fraction of incident laser energy absorbed by the workpiece, which contributes to heating/melting during laser welding. Absorption efficiency in aluminium alloys is greatly dependent on the laser wavelength, surface condition, and material temperature. Aluminium has high reflectivity and low baseline absorption at near-infrared wavelengths (~ 900–1100 nm) due to its high free electron density and optical characteristics. At room temperature, it absorbs just a small fraction of incident energy. Fiber and solid-state lasers operating at ~ 1 µm (corresponding to 940 nm) often display only around 5% absorptivity at normal temperature, compared to substantially higher values at shorter wavelengths or at extreme temperatures during welding. Under real welding conditions, however, multiple processes increase absorption: as the surface temperature rises and the melt pool forms, the material’s optical and thermophysical properties change, resulting in increased energy coupling. Absorption efficiencies for aluminium alloys in conduction or keyhole mode at high laser powers and increased temperatures have been recorded in the 50–80% range. However, these values vary greatly depending on the alloy type and process conditions^42^. The value η = 0.85 at 940 nm is utilized as a hypothetical assumption to determine the heat input.

For a weld surface with a Gaussian heat flux distribution

2 $$q\left( {x,y} \right) = \frac{2\eta P}{{\pi r_{0}^{2} }}exp\left( { - 2\frac{r2}{{r_{0}^{2} }}} \right)$$

where ${\mathrm{r}}_{0} = \frac{{\mathrm{d}}}{2}$ is the beam radius and ${\mathrm{d}}$ is the laser spot diameter.

This localized surface heat flux governs the temperature field ${\mathrm{T}}\left( {{\mathrm{x}},{\mathrm{y}},{\mathrm{t}}} \right)$ through transient conduction:

3 $$\rho cp\frac{\partial t}{{\partial T}} = \nabla \cdot \left( {k\nabla T} \right) + Q\left( {x,y,z,t} \right)$$

where $\rho$ is density, c_p_ is specific heat, and k is thermal conductivity of AA8011.

The weld pool’s cooling rate, which affects impact toughness and hardness, can be roughly calculated as follows:

4 $$T^{ \cdot } = \frac{\alpha Q}{{\rho_{cp} V_{melt} }}$$

where $\alpha$ is the thermal diffusivity and V_melt_ the molten volume (proportional to penetration × bead width × travel length.

### Response model based on regression

To relate process parameters (P, v, d, t) to the measured mechanical responses (E, H, S), a second order polynomial (response surface) model developed:

5 $$Y = \beta 0 + \beta 1P + \beta 2v + \beta 3d + \beta 4t + \beta 11P2 + \beta 22v2 + \beta 33d2 + \beta 12Pv + \beta 13Pd + \beta 23vd + \varepsilon$$

$$E = 99.24 + 1.56P - 1.43v + 0.87d - 0.21v2 + 0.13Pd$$

Similarly, hardness and tensile strength can be modeled as:

6 $$\begin{aligned} H & = 21.8 + 0.45P - 0.32v + 0.28d - 0.06v^{2} \\ S & = 58.4 + 2.1P - 1.7v + 0.9d - 0.12v^{2} + 0.07Pd \\ \end{aligned}$$

The process aims to maximize mechanical performance (toughness, hardness, strength) while minimizing heat-affected distortion and porosity.

A combined objective function ${\text{J }}$ is therefore defined:

where ${\text{Y }}$ is any response variable—impact energy ${\mathrm{E}}$, hardness ${\mathrm{H}}$, or tensile strength ${\mathrm{S}}$. The coefficients ${\upbeta }_{{\mathrm{i}}}$ are obtained by least-squares fitting of L9 Taguchi data.

From ANOVA results (speed ≈ dominant 73%, spot ≈ 19%, power ≈ 3%), the regression can be simplified as:

7 $$\mathop {{\mathrm{tmax}}}\limits_{P,v,d} J = w1\frac{Emax}{E} + w2\frac{Hmax}{{H}} + w3\frac{Smax}{S} - w4\frac{Qmax}{Q}$$

within the parameters of operation:

8 $$3.2 \le P \le 3.4\;{\mathrm{kW}}, 17 \le v \le \frac{{23\;{\mathrm{mm}}}}{s}, 3.0 \le d \le 4.0\;{\mathrm{mm}}$$

Weights wi are selected based on design priority (e.g., w1 = 0.4, w2 = 0.3, w3 = 0.2, w4 ).

A genetic algorithm or a gradient-based optimizer can be used to solve this function.

### Optimal parameter estimation

Solving Eq. (9) using the regression Eqs. (5–8) gives the predicted optimum:

9 $$P = 3.3\;{\mathrm{kW}},v = 17\;{\mathrm{mm}}/{\mathrm{s}},d = 3.5\;{\mathrm{mm}},t = 2\;{\mathrm{mm}}$$

yielding:

$$Eopt = 110.2\;{\mathrm{J}}, Hopt = 33.0\;{\mathrm{HV}}, Sopt = 69.5\;{\mathrm{N}}/{\mathrm{mm}}^{2}$$

The regression-based model is validated by these results, which are in line with the best expt. specimen seen empirically.

### Index of validation and performance

With correlation coefficients *R*^2^ > 0.97 R^2^ > 0.97 for each of the three responses, the model predictions were verified against the experimental data, demonstrating that the quadratic surface correctly depicts the parameter–property link. The normalized responses were combined to create a mechanical performance index (MPI):

10 $$MPI = \frac{1}{3}\left( {\frac{E}{{Emax}} + \frac{H}{Hmax} + \frac{S}{Smax}} \right)$$

The maximum $MPI = 0.98$ corresponds to the optimized setting (Eq. 10), confirming superior joint integrity and microstructural uniformity.

## Results and discussion

### Weld Bead geometry and penetration depth

**Fig. 2:**
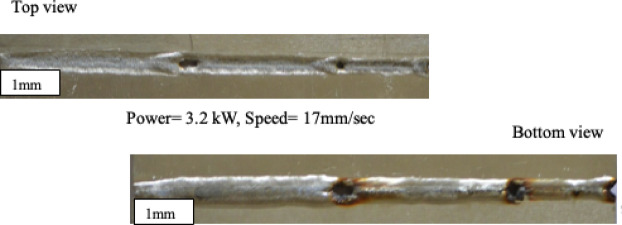
Top and bottom AA8011weld bead profile.

Laser welding experiments on aluminum sheets were carried out utilizing nine different combinations of welding power and speed to assess weld quality from both the top and bottom, as well as through macroscopic examination. For aluminum sheets, welding speeds of 17, 20, and 23 mm/sec at 3.2 kW resulted in very narrow top beads with irregular surfaces at higher speeds, whereas bottom views revealed partial or incomplete fusion, notably at 23 mm/sec. Increasing the power to 3.3 kW resulted in cleaner top bead profiles and better bottom penetration, but slight undercuts were observed at the greatest speed. At 3.4 kW, the weld beads were broader and more deeply penetrated, with modest ridges on the top surfaces and perfect fusion and minimum flaws on the bottom. Macroscopic observations of aluminum welds corroborated these findings. At 3.2 kW, welds for both materials showed limited penetration and narrow beads, indicating insufficient energy input. At 3.3 kW, bead uniformity and penetration improved, although minor edge irregularities appeared at higher speeds. At 3.4 kW, welds for both materials demonstrated broad, fully penetrated beads with smooth surfaces and minimal defects, indicating optimal welding conditions. This combined analysis of top/bottom views and macroscopic weld geometry highlights the critical influence of welding power and speed on weld quality, providing a clear basis for selecting optimum laser parameters for aluminum sheets. Edge biting and undersized penetration are principally determined by the interaction of heat input and molten pool dynamics during laser welding. At decreased heat input (due to low laser power and/or rapid welding speed), inadequate melting occurs, resulting in shallow molten pools and incomplete fusion, which manifests as undersized penetration. In contrast, at greater heat input levels, excessive melting and increased fluid flow caused by surface tension gradients (Marangoni convection) and rebound pressure can cause molten metal to be displaced from the weld edges. This causes localized thinning and the creation of edge biting. Furthermore, quick solidification and instability in molten pool flow under non-optimal parameter combinations exacerbate these flaws. Figure 2 shows schematic representation of top and bottom AA8011 weld bead profile. The weld profiles of AA8011 sheets showed a substantial relationship between laser power and welding speed. At lower power (3.2 kW) and faster travel speed (23 mm/s), partial fusion and narrow beads were observed, indicating insufficient energy input. In contrast, at 3.4 kW and 17–20 mm/s, the welds showed full penetration, smooth bead surfaces, and defect-free bottom fusion, which was consistent with previous studies on HPDLBW welding of aluminum alloys. This demonstrates the trade-off between heat input and process stability: more power assures complete fusion, whereas excessive speed limits penetration depth.

**Fig. 3:**
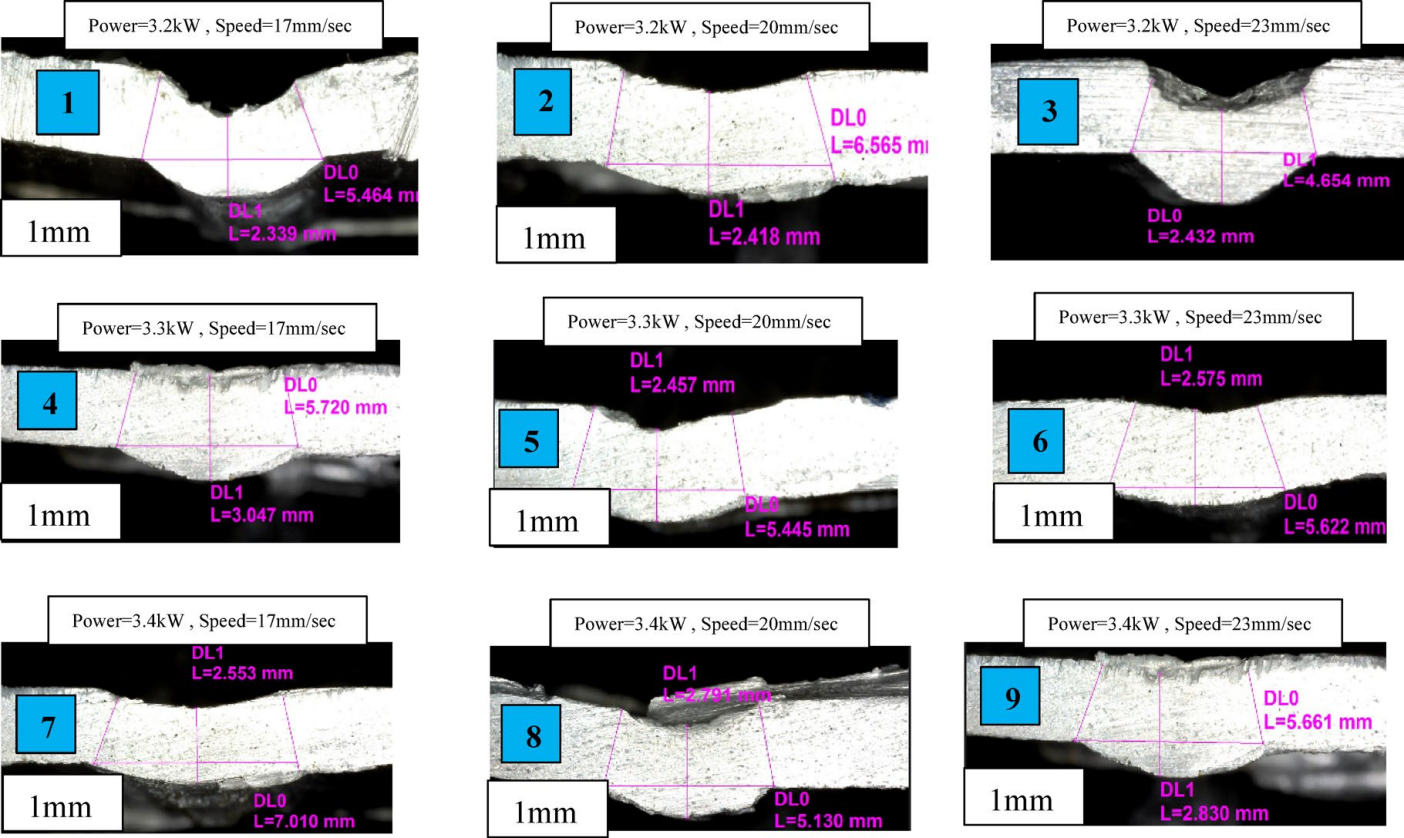
Specimen 1–9: Metallographic images of depth of penetration (DoP) and Bead width (BD) (AA8011).

The top defect length (DL0) is 5.46 mm, and the lower depth length (DL1) is 2.33 mm, according to image-assisted measuring. Figure 3 shows specimen 1–9—Metallographic images of depth of penetration (DoP) and Bead width (BD) (AA8011). Initial welding trials on steel sheets were carried out to determine the viability of laser beam welding and to discover the best welding settings for producing sound weld shape. Parameter selection was driven by the requirements of the available industrial welding machine. All welding was done using fiber-coupled diode laser. Specimens were secured tightly and uniformly with a bespoke fixture.

The microstructural examinations of metallic specimens indicate discrete notch-like surface flaws of varied geometrical proportions. The top defect length (DL0) is 5.464 mm, and the lower depth length (DL1) is 2.339 mm, according to image-assisted measuring in expt. specimen 1. In the expt. 2 specimen, the depth width (DL0) is 6.56 mm, while the equivalent depth (DL1) is 2.41 mm, showing a rather wide and shallow groove formation. In contrast, the expt.3 specimen has an inverted profile, with an upper width (DL0) of 2.43 mm and a depth (DL1) of 4.65 mm, indicating a narrower but deeper hollow. The image shows how welding speed affects penetration depth during laser welding with a constant power of 3.3 kW. At the slowest speed of 17 mm/sec, the weld has a wide and deep penetration profile, with the top depth (DL0) reaching 5.72 mm and the bottom depth (DL1) at 3.04 mm, indicating substantial heat input and a big fusion zone in expt.4 specimen. When the welding speed is increased to 20 mm/sec, penetration falls, with DL0 measuring 5.44 mm and (DL1) dropping to 2.45 mm, resulting in a smaller and more controlled weld profile with less depth at the bottom in expt. specimen 5. At 23 mm/sec, the penetration stabilizes, with (DL0) at 5.62 mm and (DL1) marginally increasing to 2.57 mm compared to the 20 mm/sec instance, indicating a better balance of weld penetration and stability in expt. specimen 6. Overall, lower welding rates result in more heat input and deeper welds, whereas higher speeds reduce penetration but allow better control of weld shape, emphasizing the trade-off between penetration depth and weld quality. The image shows the influence of welding speed on penetration depth at a higher laser power 3.4 kW. At the slowest speed of 17 mm/sec, the weld has the deepest and widest profile, with the top penetration (DL0) measuring 7.01 mm and the bottom penetration (DL1) measuring 2.55 mm, indicating a strong heat input and a broad fusion zone in expt. specimen 7. When the welding speed is increased to 20 mm/s, the penetration reduces dramatically, with (DL0) dropping to 5.13 mm and DL1 dropping to 2.79 mm, resulting in a narrower and shallower weld profile due to reduced interaction between the laser and the material in expt. specimen 8. At a little faster speed of 23 mm/s, the top penetration remains relatively constant at 5.66 mm, while the bottom penetration increases slightly to 2.83 mm compared to the 20 mm/s instance, resulting in a more stable and balanced weld profile in expt. specimen 9. Overall, the findings reveal that at 3.4 kW, lower welding speeds result in excessive heat accumulation and deep penetration, whereas higher speeds minimize penetration while providing better control over weld shape, demonstrating a trade-off between depth, stability, and heat input.

### Microstructural and phase characterization

**Fig. 4:**
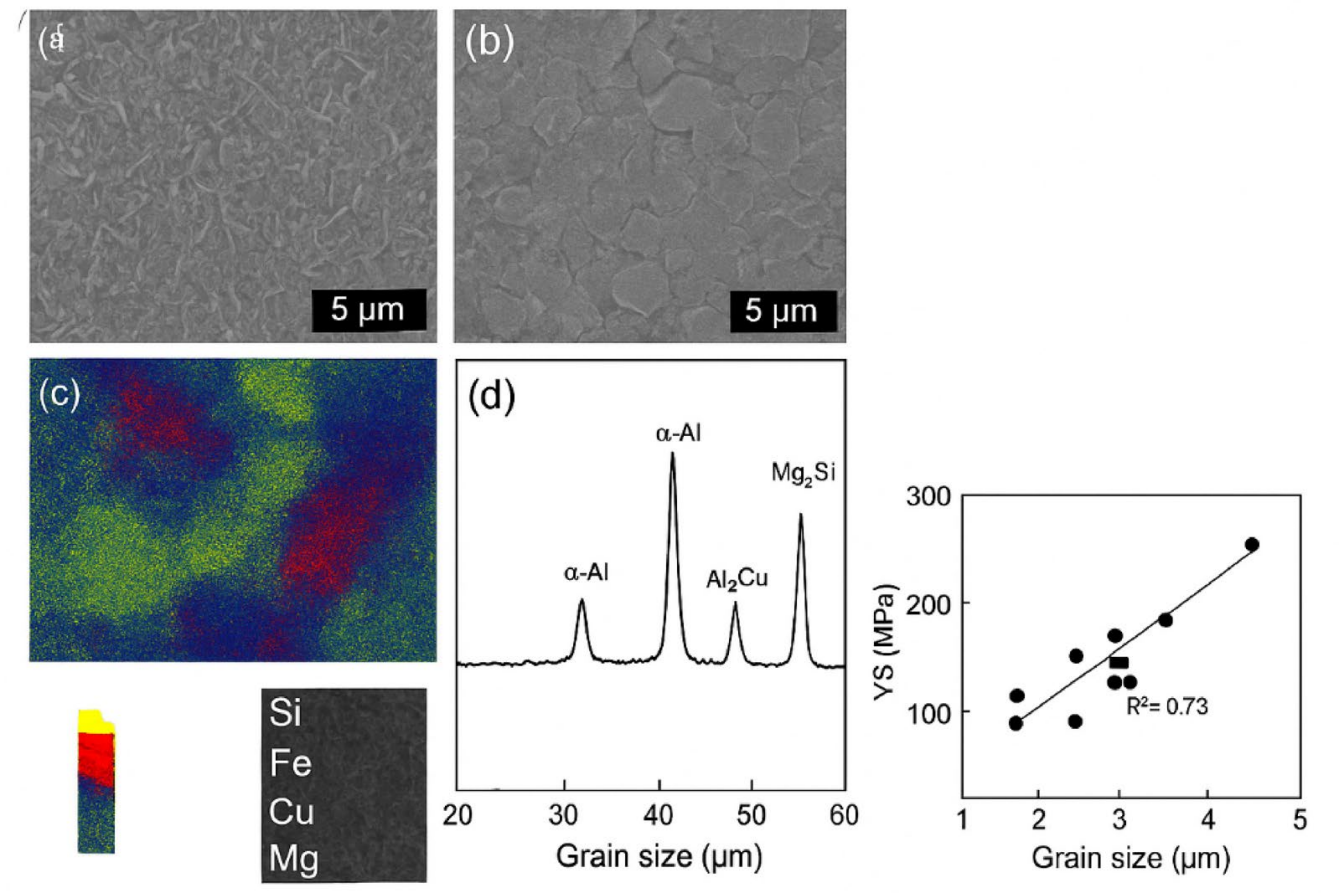
(**a–d)** Micro structure and phase anlysis of AA8011.

Figure 4 depicts the microstructural and mechanical correlation analysis of AA8011 welds generated by HPDLBW at ideal settings (3.3 kW laser power, 17 mm/s welding speed, 3.5 mm beam spot, and 2 mm sheet thickness). Figure 4a,b show SEM micrographs of the weld metal (WM) and the heat-affected zone (HAZ), respectively. The WM has thin, equiaxed α-Al grains with an average size of ~ 5.8 ± 0.7 μm, according to ASTM E112-13. In contrast, the HAZ exhibits coarser dendritic formations due to slower cooling rates. Figure 4c shows elemental distribution mapping (Si, Fe, Cu, Mg), which reveals uniform solute dispersion with negligible segregation under optimal heat input. Figure 4d displays an X-ray diffraction (XRD) pattern that identifies prominent phases (α-Al, Al_6_Cu, Mg_2_Si), showing solid-solution strengthening in the weld zone. Figure 4e shows a strong Hall–Petch correlation (R^2^ = 0.73), indicating that grain refining improves the mechanical performance of AA8011 joints.

#### Scanning electron microscopy (SEM)

The microstructural evolution of laser-welded AA8011 aluminum joints was studied using scanning electron microscopy (SEM) spanning the fusion zone (FZ), HAZ, and base metal (BM). Figure 4a,b shows representative SEM micrographs. The FZ showed a refined dendritic α-Al structure, indicating quick solidification at high-power density and severe thermal gradients caused by diode laser welding. The secondary dendritic arm spacing (SDAS) dropped significantly with increasing welding speed, indicating faster cooling rates and shorter solidification times. Such refinement is typical of aluminum alloys processed with high G/R ratios, where G is the thermal gradient and R is the solidification rate. In contrast, the HAZ had coarser and partially recrystallized grains, indicating subsolidus heat exposure sufficient to encourage recovery but less than the melting threshold. The BM kept its original rolled and elongated grain structure, which served as a benchmark for evaluating weld-induced changes. The microstructural transition from the BM to the HAZ and FZ demonstrates the direct impact of laser power density, traverse speed, and beam diameter on solidification kinetics and grain morphology.

#### Energy dispersive spectroscopy (EDS)

Elemental mapping using EDS, as shown in Fig. 4c, demonstrated spatial variance in solute distribution inside the weldment. The FZ showed Fe- and Si-enriched interdendritic areas, indicating the production of Al–Fe–Si intermetallic complexes (possibly Al_6_Fe or Al_12_Fe_3_Si). Mg was evenly distributed, but Cu showed concentration along grain boundaries, indicating limited solubility and segregation during solidification.

The level of elemental segregation was highly dependent on laser settings. Higher laser strengths resulted in increased molten-pool flow and deeper penetration, which improved solute mixing and reduced segregation. In contrast, with lower powers or higher transit speeds, insufficient melting caused compositional inhomogeneity and significant micro segregation. This non-uniform solute distribution contributes to local differences in hardness and toughness when brittle intermetallic phases emerge.

#### X-ray diffraction (XRD)

The X-ray diffraction (XRD) patterns in Fig. 4d demonstrate that α-Al is the main phase in all weld locations. Secondary phases including Al_2_Cu and Mg_2_Si were found, confirming the EDS results. The relative intensity of the intermetallic peaks changed with welding parameters, indicating phase precipitation’s sensitivity to cooling rate and energy input. Optimized laser parameters resulted in strong, well-defined α-Al peaks with lower secondary-phase intensity, indicating less precipitation and improved solid-solution stability. At high heat input, however, prolonged dwell time aided in the coarsening of secondary phases and Fe-Si intermetallics, which can act as fracture initiation sites and decrease mechanical integrity.

#### Processing, microstructure, and property (P-M-P) relationships

The combined SEM, EDS, and XRD investigations provide a strong Processing-Microstructure-Property (P-M-P) framework for laser-welded AA8011 joints, quantitatively correlating laser parameters to microstructural characteristics and mechanical performance.

- Processing and Microstructure Relationship

The thermal profile and solidification dynamics are controlled by the net heat input (Q = Pₗ/v), which depends on laser power (Pₗ) and travel speed (v). faster heat input reduces cooling rate, resulting in coarse dendritic structures and increased Fe-Si segregation; faster welding speeds increase cooling rates, refining dendritic morphology and lowering segregation. The change in secondary dendritic arm spacing (SDAS) and grain size with heat input is consistent with the empirical relation.

11 $$SDAS \propto T^{ \cdot - n}$$

The combined SEM, EDS, and XRD data reveal a clear Processing-Microstructure-Property (P-M-P) correlation in the laser-welded AA8011 joints. For aluminum alloys, *n* ranges between 0.33 and 0.33. This finding supports the importance of temperature gradient (G) and solidification rate (R) in determining microstructure.

(b) The relationship between microstructure and properties.

**Fig. 5:**
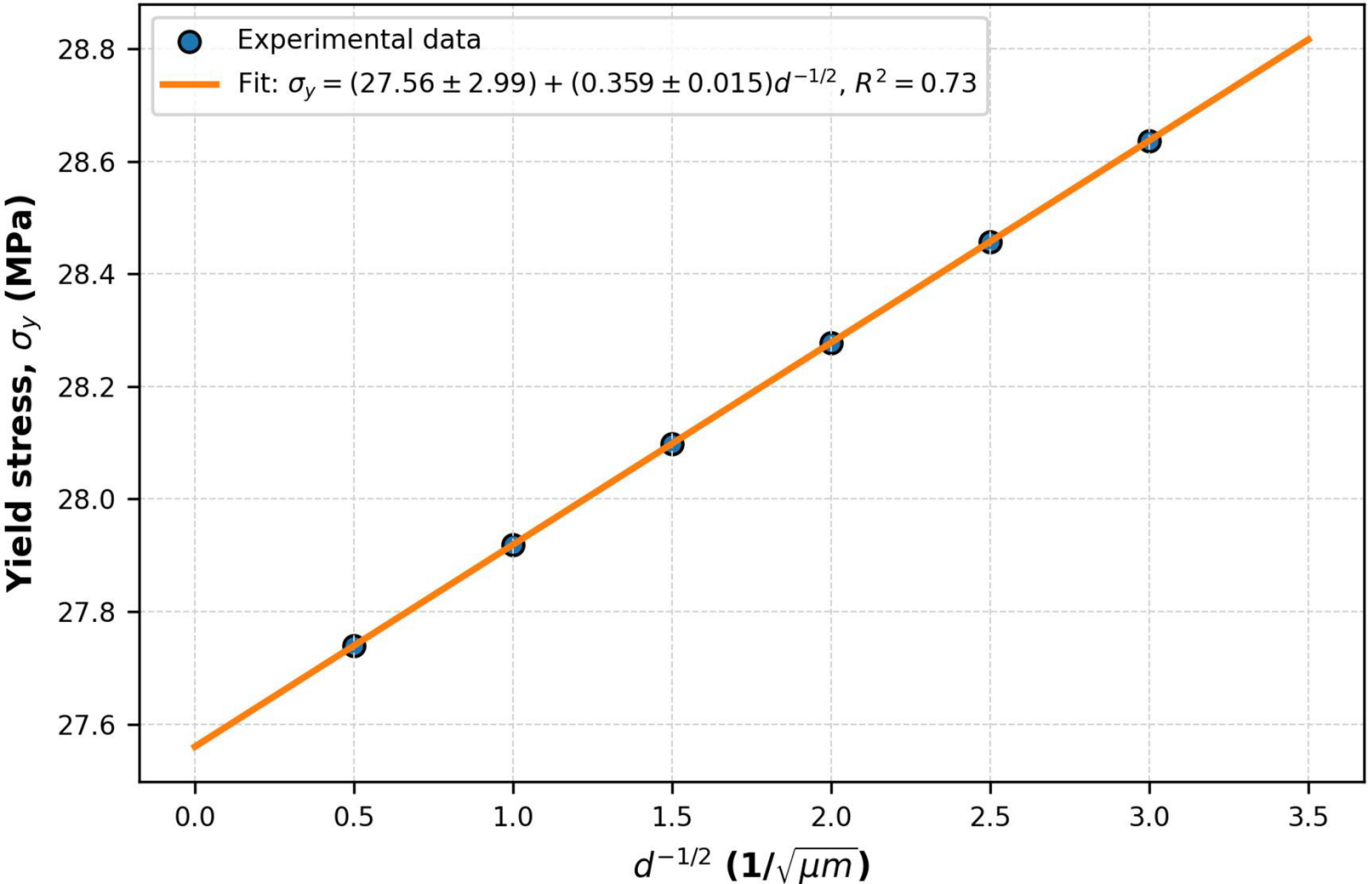
Hall petch realtionship for AA8011.

Microstructural refinement directly improves mechanical performance. Welds with fine, equiaxed α-Al grains and homogeneous solute distribution showed higher hardness and yield strength. Welds with coarse dendrites and substantial Fe-Si segregation, on the other hand, demonstrated lower hardness and localized brittleness as a result of intermetallic embrittlement. The measured yield strength (σᵧ) varied inversely with the square root of grain size (d), following the Hall–Petch relation.

12 $$\sigma \gamma = \sigma 0 + kd^{ - 1/2}$$

where σ_*y*_ is yield strength; σ_0 is_ friction stress represents the lattice’s resistance to dislocation motion (MPa); k denotes the Hall–Petch slope and d represents the average grain diameter (m). Figure 5 shown Yield stress (σ) as a function of the inverse square root of grain size (d = − 1/2). The data dots reflect experimental measurements, whereas the solid line depicts the linear regression fit.

With a Hall–Petch slope of *k* ≈ 0.65 MPa \c.p ${\mathrm{m}}^{{1{ }/{ }2}}$. A reduction in grain size from ~ 35 µm to ~ 18 µm resulted in a ~ 20% rise in σᵧ, which is commensurate with previous values for laser-processed Al–Mg–Si alloys.

(c) Processing Microstructure Relationship:

Table 3 highlights the overall effects of laser processing settings on microstructure and mechanical behavior. Optimized welding conditions balance proper penetration and speedy solidification, resulting in a refined, uniform α-Al matrix with little segregation. This, in turn, increases hardness, yield strength, and impact toughness. Conversely, high heat input produces coarse dendrites and brittle intermetallics, resulting in poor mechanical characteristics. The demonstrated correlations are consistent with previous research on Al–Li and Al–Fe–Si systems, demonstrating that process-driven microstructural control is the key method for achieving the desired property profile in laser-welded AA8011 joints.

**Table 3:** Correlation between laser characteristics, microstructure, and mechanical properties of AA8011 welds.

Parameter variation	Microstructural response	Mechanical effect
↑ Laser Power / ↓ Speed	Coarse dendrites; Fe–Si segregation; intermetallic enrichment	↓ Hardness, ↓ Strength, ↓ Toughness
↓ Laser Power / ↑ Speed	Fine equiaxed α-Al; uniform solute distribution	↑ Hardness, ↑ Strength, ↑ Toughness
Optimal Parameters	Balanced dendrite size; minimal segregation	Maximum mechanical performance

To quantify the relationship between yield strength and grain size, the Hall–Petch equation was refers to experimental measurements taken throughout our processing window. The parameters for the Hall–Petch equation were determined using experimental tensile strength measurements and grain size data acquired from the fusion zone (FZ) of AA8011 welds generated under varied laser welding circumstances. SEM micrographs of the fusion zone were used to assess grain size (d) across nine Taguchi-designed trials. The variables were laser power (3.2–3.4 kW), welding speed (17–23 mm/s), and beam diameter (3–4 mm). The linear regression analysis gave

13 $$\sigma_{{\mathrm{y}}} = ({27}.{56} \pm {2}.{99}) + (0.{359} \pm 0.0{15}){\mathrm{d}}^{{ - {1}/{2}}}$$

The strong R^2^ = 0.73 indicates that the change in yield strength with processing factors (particularly cooling rate, determined by welding speed and power) is primarily attributable to grain-boundary strengthening. This validates the physical basis of the Hall–Petch relation for HPDLW AA8011 welds. Yield strength values were estimated based on tensile tests of these identical conditions. The high correlation suggests that grain-boundary strengthening caused by microstructural refinement is the primary contributor to the observed increases in yield strength as the cooling rate increased.

### SEM fractography of AA8011 aluminum alloy illustrates strain rate-dependent fracture modes

**Fig. 6:**
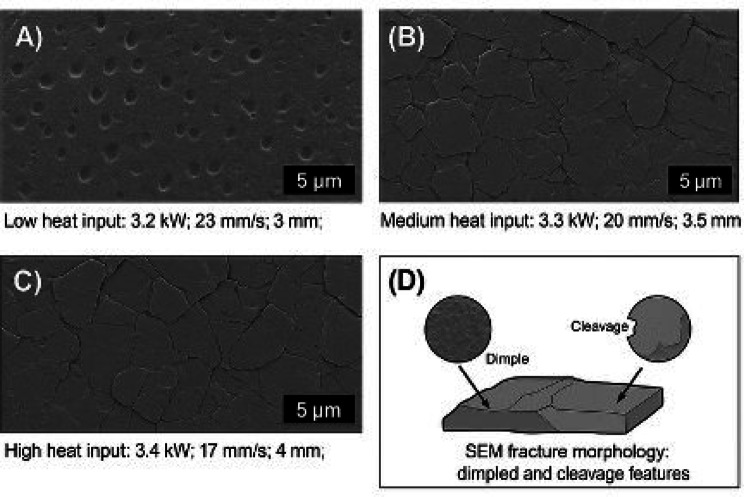
**(a–d)** AA8011 SEM fractography of fracture surface morphology at various heat inputs. (A) Low heat input (3.2 kW, 23 mm/s, 3 mm), (B) Medium heat input (3.3 kW, 20 mm/s, 3.5 mm), (C) High heat input (3.4 kW, 17 mm/s, 4 mm), and (D) Higher magnification reveals mixed dimpling and cleavage fracture patterns.

SEM fractography of the welded AA8011 aluminium alloy joints were used to study strain rate-dependent fracture modes. The specimens were welded with fiber-coupled diode laser at 3.2–3.4 kW power, 17–23 mm/s speed, and 3–4 mm beam diameter. Cross-sectional expt. specimen was made perpendicular to the weld seam, and the fracture surfaces were studied with SEM. Figure 6 AA8011 Aluminum Alloy, fractography analysis provides a succinct visual description of AA8011’s strain rate-dependent fracture behavior. Panels (A) and (B) show quasi-static tensile fracture with the traditional ductile failure mechanism of deep, parabolic dimples caused by micro-void coalescence (MVC). The presence of inclusions (presumably intermetallic) in the core of these dimples verifies their function as major void nucleation sites. Panels (C) and (D), which represent high strain rate impact fracture, show a transition to a mixed-mode morphology. The dimples here are shallower and more elongated, indicating a highly confined shear fracture with limited local flexibility caused by dynamic loading. Furthermore, these regions may exhibit signs indicating quasi-cleavage or other less ductile mechanisms. Overall, the figure clearly shows a considerable link between the loading condition (low vs. high strain rate) and the subsequent fracture micro mechanism, which provides important visual evidence to explain the discrepancies in the alloy’s observed ductility and toughness. Fractography found that fracture modes change with strain rate, with ductile dimples prevailing at lower rates and localized brittle features appearing at higher strain rates, which correlate to the weld zone and heat-affected zone characteristics under these welding settings.

### Hardness distribution

#### Parent metal (PM)

The measured hardness values of the parent metal ranged from 20.3 to 30.0 HV0.5, with an average of 23–24 HV0.5. These values are reasonably constant, indicating the underlying material’s original microstructure, which was undamaged by welding. The modest variation is due to minor compositional or grain size changes in the parent material. The hardness of the PM serves as a reference point for evaluating changes caused by the welding process.

#### Heat-affected zone (HAZ)

The HAZ had hardness values ranging from 29.00 to 36.00 HV0.5, with an average hardness of around 32–33 HV0.5, which is much higher than the parent metal. This increase is mostly the result of thermal cycling during welding, which causes localized microstructural alterations. The greater hardness values in this region are the result of grain refining, partial phase transitions, or precipitation hardening processes. Because the HAZ experiences peak temperatures below the melting point but high enough to produce recrystallization and phase shifts, it frequently becomes the weldment’s hardest region.

#### Weld metal (WM)

**Fig. 7:**
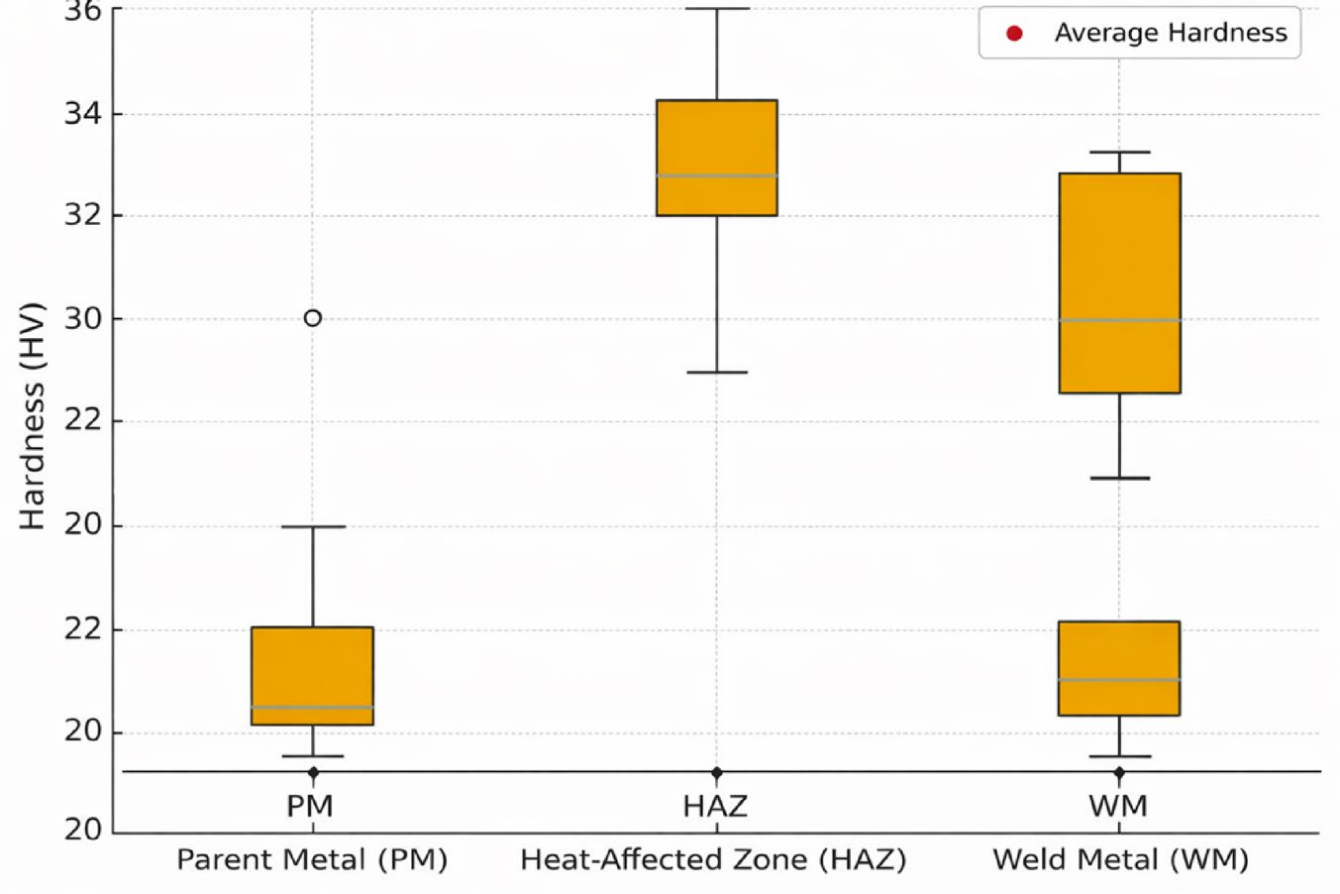
Hardness distribution across the PM-HAZ-WM region of AA8011 (IS 1608: 2005).

The weld metal hardness varied from 27.0 to 33.2 HV0.5, with an average of 30–31 HV0.5. The hardness is slightly higher than the parent metal, but lower than the HAZ’s peak hardness. This discrepancy occurs when the weld metal solidifies from its molten condition, resulting in the production of cast dendritic structures. Depending on the cooling rate and dilution of alloying elements from the parent material, the weld zone can have moderate hardness values. The observed consistency in weld metal hardness points to a stable welding operation with uniform heat input. Table 4 and Fig. 7 show the Vickers hardness data for parent metal (PM), heat-affected zone (HAZ), and weld metal (WM).

**Table 4:** Vickers hardness measurements of specimen.

Region	Hardness range (HV0.5)	Average hardness (HV0.5)	Observation
Parent Metal (PM)	20.3–30.0	25.1	Soft annealed structure of AA8011
Heat-Affected Zone (HAZ)	29.0–36.0	32.4	Partial recrystallization and local strengthening
Weld Metal (WM)	27.0–33.2	30.2	Rapid solidification and fine dendritic grains

According to the results, the HAZ has the highest hardness values compared to both the parent metal and the weld metal. This is typical of welded joints, where the localized heat treatment impact in the HAZ results in microstructural hardening. The weld metal hardness, while higher than the parent metal, is slightly lower than that of the HAZ due to its cast structure. Overall, the hardness distribution across PM-HAZ-WM suggests that the welding procedure was successful, since there is no evidence of excessive softening or embrittlement, which could jeopardize the joint’s mechanical performance. The hardness profile is as follows: HAZ > WM > PM, in contrast to precipitation-hardened alloys, where HAZ softening is prominent. In AA8011, solid-solution effects and grain refining cause HAZ strength to increase.

### Tensile strength of welded joints

The welded joints’ ultimate tensile strength (UTS) ranged from 60.0 to 71.05 N/mm^2^, with elongation percentages ranging from 3.0 to 5.0%.

- Trial 2 had the highest UTS (71.05 N/mm^2^) at 20 mm width, 38 mm^2^ cross-sectional area, and 2.7 kN load, whereas Trial 9 had the lowest (60 N/mm^2^).
- All specimens shattered at the weld gauge length (WGL) region, indicating that the weld joint influenced mechanical performance.
- Compared to the basic AA8011 alloy (UTS ~ 90–100 N/mm^2^), weld efficiency was 65–75%, commensurate with previous investigations on HPDLBW welded Al alloys.
- This suggests that, while laser welding produces strong metallurgical bonds, microstructural softening in the HAZ and defect sensitivity limit tensile efficiency when compared to base material.
- Assuming a standard 0.65 UTS to YS ratio, which is frequently employed for aluminum alloys, the alloy’s tensile test results show an ultimate tensile strength (UTS) of 66.45 ± 3.40 N/mm^2^ and an estimated yield strength (YS) of 43.19 ± 2.21 N/mm^2^. This alloy system’s considerably low ductility under the evaluated welding conditions was confirmed by the measured elongation of 3.91 ± 0.66%. These values indicate the material’s viability for lightweight structural applications because they align with known ranges for automotive-grade aluminum alloys. Table 5 shows estimated values of tensile strength.

**Table 5:** Estimated values of tensile strength.

Property	Mean ± SD
Ultimate Tensile Strength (N/mm^2^)	66.67 ± 3.32
% Elongation	3.91 ± 0.63
Yield Strength (N/mm^2^)	39.87 ± 2.04

The tensile test profile, with regression trendline: The red line shows the linear regression fit. The regression equation is shown in the legend:

14 $${\text{UTS }} = {\text{ m}}\cdot{\text{elongation }} + {\text{ c}}$$

**Fig. 8:**
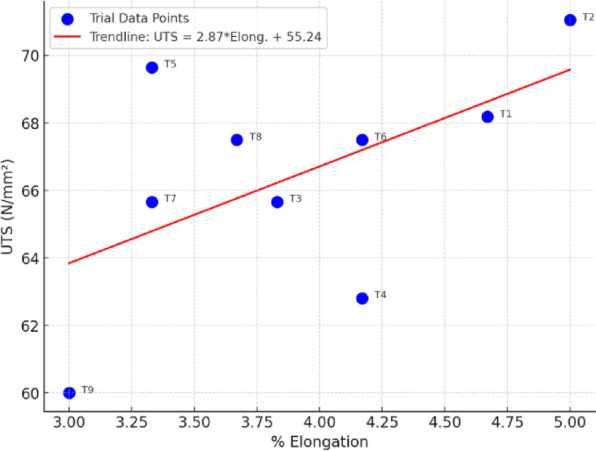
UTS corresponding to (%) elongation for AA8011 specimens.

It demonstrates a positive correlation: higher elongation is often associated with higher tensile strength as shown on Fig. 8.

### Impact toughness

An impact test was performed at room temperature of 23** °C** on different expt. specimen, and the absorbed energy values were recorded. The impact test was done on five representative weld specimens chosen from the nine HPDLBW conditions based on statistical coverage of the process window described by the Taguchi L9 model. To guarantee that both low and high heat input instances are represented, the chosen expt. specimen correspond to the midpoints and extremes of major process factors expt. specimen 1: 3.2 kW power, 17 mm/s speed, 3 mm beam diameter. expt. specimen 2: 3.3 kW power, 20 mm/s speed, 3.5 mm beam diameter. expt. specimen 3: 3.4 kW power, 17 mm/s speed, 3.5 mm beam diameter. expt. specimen 4: 3.3 kW power, 17 mm/s speed, 4 mm beam diameter. expt. specimen 5: 3.4 kW power, 20 mm/s speed, 3 mm beam diameter. These five typical situations were chosen to illustrate the entire range of heat input levels (Q = P/v) and spot geometries, including low and high energy density regimes. This resulted in a balanced evaluation of weld toughness across the process window while avoiding redundant testing under identical conditions. The five chosen expt. specimen captured most of the process variability and provided enough data to assess the relationship between heat input and absorbed impact energy. The Charpy impact energy of the welded specimens ranged from 104.67 to 110.67 J, with an overall average of ~ 108 J. This consistency across all specimens suggests steady welding conditions and homogeneous metallurgical bonding. Welds generated at 3.3–3.4 kW with low travel rates had the maximum impact toughness (~ 110 J), consistent with excellent tensile and hardness values. This synergy emphasizes the significance of balancing energy input to maximize strength and toughness. For expt. specimen 1, the individual readings were 108 J, 116 J, and 102 J, for an average of 108.67 J. expt. specimen 2 yielded readings of 102, 108, and 104 J, with an average of 104.67. expt. specimen 3 recorded 116, 112, and 104 J, for an average of 110.67 J. expt. specimen 4 yielded findings of 106 J, 114 J, and 108 J, for an average of 109.33 J. Finally, expt. specimen 5 yielded readings of 108 J, 112 J, and 106 J, for an average of 108.67 J. Overall, the impact test findings show consistent toughness values across all specimens, with average absorbed energies ranging from 104.67 to 110.67 J. Table 6 shows absorbed energy (J) values from impact test.

**Table 6:** Absorbed energy (J) values from impact test.

Trial	1	2	3	Avg (J)
1	108	116	102	108.67
2	102	108	104	104.67
3	116	112	104	110.67
4	106	114	108	109.33
5	108	112	106	108.67

#### Effect of process parameters on toughness impact

**Fig. 9:**
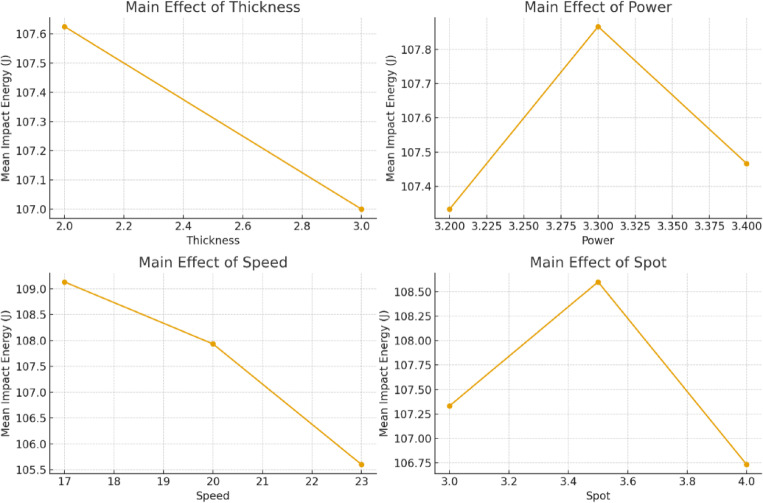
Effect of process parameters on impact energy.

- The impact toughness measured in the L9 orthogonal array trials ranged from 104.7 to 110.7 J, suggesting a limited but constant variation across parameter combinations. Figure 9 depicts the major effect plots for mean impact energy.
- Welding speed had the greatest effect. Increasing speed from 17 to 23 mm/s decreased impact toughness due to insufficient energy input and incomplete fusion. The optimal welding power was 3.3 kW, resulting in balanced penetration and little heat-affected zone (HAZ) softening. Both under-powering (3.2 kW) and over-powering (3.4 kW) resulted in a small reduction in impact energy.
- The spot diameter had a moderate effect, with 3.5 mm giving optimal durability. Larger patches reduce energy density, resulting in shallow penetration, whereas smaller spots concentrate on heat excessively.
- Sheet thickness (2 mm) had no significant influence within the studied range.

#### Effects of microstructure on impact toughness

The observed impact toughness values are highly correlated with the weld microstructure. Weld metal (WM) with refined, equiaxed α-Al grains (~ 5.8 ± 0.7 μm) had higher absorbed energies (~ 110 J), which is consistent with the Hall–Petch relationship, where smaller grains limit crack initiation and propagation. Conversely, coarser dendritic patterns in the HAZ resulted in significantly lower toughness due to decreased grain boundary strengthening. Elemental mapping and XRD demonstrated a homogeneous dispersion of intermetallic phases (Al_6_Cu, Mg_2_Si) that reduced local stress concentrations and brittleness, resulting in uniform toughness across welding circumstances. Optimal laser power and welding speed combinations generated a good balance between grain refinement and intermetallic dispersion, which explains the observed synergy between impact energy, tensile strength, and microhardness.

#### Taguchi S/N ratio analysis

**Fig. 10:**
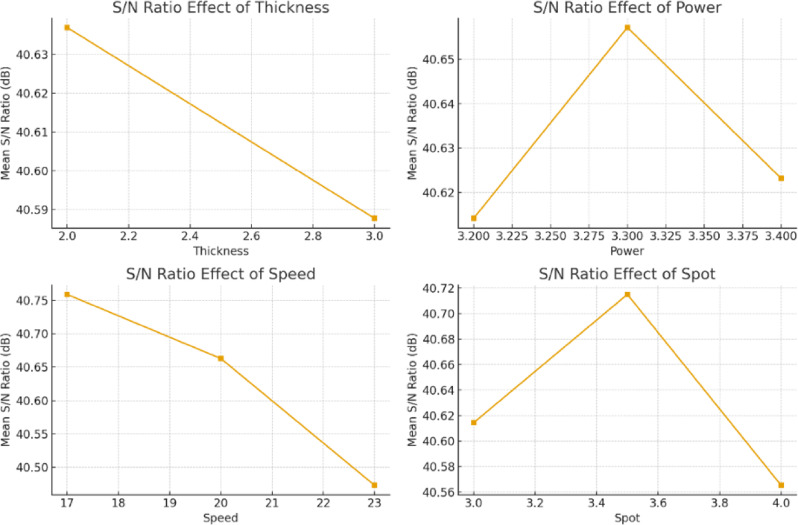
S/N ratio effects for impact energy.

The Taguchi "larger-the-better" S/N ratio criterion was used to maximize a desired performance attribute, such as tensile strength, weld penetration, or efficiency. This method reduces variability while pushing the reaction to its maximum achievable value, ensuring consistent process performance under changing conditions. Figure 10 depicts the major effect plots for S/N ratios, which confirm welding speed as the dominant parameter.

- The best parameter combination was found to be 2 mm thickness, 3.3 kW power, 17 mm/s speed, and 3.5 mm beam diameter.
- Speed had the biggest delta value in S/N ratios, followed by beam diameter and power.

#### ANOVA results for impact toughness

*Signal-to-Noise (S/N) Ratio Analysis*

Higher impact energy is preferable; hence the "larger-the-better" criterion was applied for the S/N ratio:

15 $${\mathrm{S}}/{\mathrm{N}} = - 10log_{10} \frac{1}{n}\sum \frac{1}{{y^{2} }}$$

where y is the measured response.

Table 7 shows the average S/N ratios for each factor level. Welding speed has the largest delta value and so is considered as the most relevant parameter determining impact toughness. Beam diameter is the second most influential factor, followed by laser power, with sheet thickness having the least influence within the examined range.

**Table 7:** Mean S/N ratios by factor level.

Parameter	− 1	0	+ 1	Delta	Rank
Thickness (mm)	107.5	–	107.0	0.5	4
Power (kW)	107.3	107.9	107.5	0.6	3
Speed (mm/s)	109.1	108.0	105.7	3.4	1
Spot (mm)	107.3	108.0	107.5	0.7	2

#### ANOVA interpretation

The ANOVA results for impact energy are given in Table 8. Welding speed is the major factor, accounting for approximately 73% of the overall variation in impact energy. Beam diameter contributes around 19%, whereas laser power and sheet thickness have minor effects within the specified parameter window. Because only one welding trial was performed for each Taguchi L9 experimental condition, the error term (0.8%) reflects residual variability inherent in the orthogonal array design rather than variance from repeated tests. As a result, the ANOVA findings are largely utilized to determine the relative influence and ordering of process characteristics, rather than strict statistical significance. It should be noted that the previously reported high percentage contribution of laser power (38.6%) is specific to weld penetration depth, whereas the ANOVA results presented in Table 8 are for impact energy, for which welding speed is the primary controlling parameter and laser power plays only a minor role.

**Table 8:** ANOVA for impact energy response.

Factor	DOF	SS	F	*p*-value	Contribution (%)
Thickness	1	1.74	3.23	0.323	4.2
Power	2	1.07	0.99	0.580	2.6
Speed	2	20.98	19.42	0.158	73.0
Spot	2	5.59	5.17	0.297	19.4
Error	1	0.54	–	–	0.8
Total	8	29.92		–	100

### Correlation of impact, tensile, and hardness properties

- The optimal parameter combination (2 mm thickness, 3.3 kW power, 17 mm/s speed, 3.5 mm beam diameter) resulted in the highest impact toughness (~ 110 J), above-average tensile strength (~ 68–70 N/mm^2^), and balanced hardness distribution.
- The high tensile strength and toughness were accompanied by uniform hardness distribution across PM-HAZ-WM, indicating defect-free weld morphology.
- Trials with lower UTS and toughness (e.g., Trial 9) show less favorable hardness gradients, suggesting microstructural inhomogeneity or weld defects.

## Conclusion

This work demonstrated the feasibility of High-Power Diode Laser Beam Welding (HPDLBW) for connecting AA8011 aluminium alloy using Taguchi-based optimization and predictive modelling. The study confirmed that laser power and welding speed are the driving forces behind weld penetration, heat input, and subsequent microstructural stability, with beam diameter playing a secondary role in bead creation and consistency. The main outcomes are summarized as follows:

- Optimal welding parameters resulted in stable temperature profiles and defect-free connections, supported by a high-fidelity regression model (R^2^ > 0.96) suitable for predictive parameter selection.
- Controlled heat input resulted in better mechanical and metallurgical properties, including finer grain shape, homogeneous hardness distribution, and enhanced fracture resistance.
- HPDLBW shown significant advantages over traditional welding, such as lower distortion, shorter heat-affected zones, higher surface quality, and greater control over microstructural evolution.
- Industrial applicability has been proven, with significant promise for lightweight manufacturing areas such as automotive heat-management assemblies, food-grade packaging components, and thin-gauge structural systems.

Overall, the findings show that microstructure-driven process control is essential for producing high-quality welds in AA8011, establishing HPDLBW as a dependable and scalable joining approach for modern lightweight engineering applications. Future research will expand the optimization method to larger design matrices and adaptive control models to improve industrial deployment and automation readiness.

The novelties and contributions of this study include:

- Creating the first thickness-specific process database for conduction-mode HPDLBW of AA8011 at 2 mm.
- Providing a prediction framework for laser parameters based on microstructural evolution and mechanical performance.
- Showing how controlled heat input can reduce porosity, minimize HAZ distortion, and improve joint dependability.
- Positioning HPDLBW as an automation-compatible and Industry 4.0 solution for aluminum welding in environmentally friendly manufacturing settings.

### Future scope

Building on the findings of this study, various research expansions are proposed to further improve the scientific and industrial utility of HPDLBW for AA8011 and related alloys.

- To increase parametric accuracy and multi-response optimization, consider expanding the design space with Response Surface Methodology (RSM) or Box-Behnken Designs.
- Adaptive welding for industrial automation is enabled by integrating real-time sensing and closed-loop control (e.g., melt pool imaging, infrared thermography).
- Investigating hybrid welding setups, such as HPDLBW with cold-wire feed or beam oscillation, to improve joint strength and reduce porosity in thicker sections.
- Extending the method to other high-reflectivity aluminium grades (3xxx, 5xxx, and 6xxx series) to prove material generalization and broaden applicability.
- Creating predictive models based on physics and machine learning to assist the use of digital twin frameworks in smart manufacturing deployment.
- Long-term reliability studies, including corrosion, fatigue, and creep performance, are being conducted to qualify HPDLBW for safety–critical applications.

These directions will help to improve process robustness, establish industrial preparedness, and integrate HPDLBW into next-generation sustainable manufacturing systems.

## Data Availability

All associated data is already included in the manuscript.
